# Impact of a health education intervention on knowledge, attitudes, and use of visibility materials among commercial motorcycle riders: a quasi-experimental study in Cameroon

**DOI:** 10.1186/s12889-026-28951-4

**Published:** 2026-08-07

**Authors:** Chrisantus Eweh Ukah, Nicholas Tendongfor, Alan Hubbard, Elvis Asangbeng Tanue, Rasheedat Oke, Nahyeni Bassah, Larissa Kumenyuy Yunika, Claudia Ngeha Ngu, S. Ariane Christie, Dickson Shey Nsagha, Alain Chichom-Mefire, Catherine Juillard

**Affiliations:** 1 Department of Public Health and Hygiene, Faculty of Health Sciences, University of Buea, Buea, Cameroon; 2 Division of Epidemiology, School of Public Health, University of California Berkeley, Berkeley, CA USA; 3 Program for the Advancement of Surgical Equity, Department of Surgery, University of California Los Angeles, Los Angeles, CA USA; 4 Department of Surgery and Specialties, Faculty of Health Sciences, University of Buea, Buea, Cameroon

**Keywords:** Road traffic injuries, Motorcycle safety, Visibility materials, Health education, Quasi-experimental study, Cameroon

## Abstract

**Background:**

Commercial motorcycle riders are among the most vulnerable road users in low- and middle-income countries, with poor visibility contributing significantly to road traffic crashes, particularly under low-light conditions. Visibility materials such as reflective clothing and functional motorcycle lighting systems are effective, low-cost injury prevention measures; however, their use remains suboptimal. This study evaluated the impact of a theory-driven health education intervention on knowledge, attitudes, and use of visibility materials among commercial motorcycle riders in Cameroon.

**Methods:**

A quasi-experimental, non-randomized controlled before-and-after study was conducted in Limbe (intervention) and Tiko (control) Health Districts. Participants were drawn from a baseline cross-sectional study and followed over an eight-month intervention period. The intervention, guided by the Health Belief Model and developed using the Intervention Mapping framework, combined face-to-face sensitization sessions with mobile phone–based messaging tailored to participants’ literacy levels. Data were collected at baseline and endline using structured questionnaires and direct observation checklists. Knowledge, attitude, and practice scores were treated as continuous variables. Difference-in-differences analysis using generalized estimating equations was employed to estimate the intervention effect, adjusting for socio-demographic factors.

**Results:**

A total of 249 riders were retained at endline (149 intervention, 100 control). In the intervention group, significant improvements were observed in knowledge (mean difference = 1.75, *p* < 0.001), attitudes (mean difference = 5.60, *p* < 0.001), and practices (mean difference = 0.36, *p* = 0.012), while no significant changes were observed in the control group. Difference-in-differences analysis showed that the intervention was associated with significant increases in knowledge (β = 1.68, 95% CI: 1.16–2.20) and attitudes (β = 5.51, 95% CI: 4.22–6.80), but not practices (β = 0.34, 95% CI: −0.11–0.79). For visibility material use, the intervention was associated with a significant increase in reflective jacket use (AOR = 4.34, 95% CI: 1.88–10.02), while no significant effects were observed for other visibility materials.

**Conclusion:**

A theory-informed health education intervention significantly improved knowledge, attitudes, and reflective jacket use among commercial motorcycle riders. However, improvements in other visibility-related practices were limited, suggesting that educational strategies alone may be insufficient to address structural and resource-related barriers. Multi-level interventions combining education with improved access and enforcement are needed to achieve sustained adoption of visibility materials for injury prevention.

**Trial registration:**

ClinicalTrials.gov Identifier: NCT07087444. Registered on July 28, 2025.

**Supplementary Information:**

The online version contains supplementary material available at https://doi.org/10.1186/s12889-026-28951-4.

## Background

Road traffic injuries (RTIs) remain a major global public health challenge, causing approximately 1.19 million deaths each year and representing one of the leading causes of mortality and disability worldwide, particularly among young people and populations living in low- and middle-income countries (LMICs) [1]. Motorcyclists are among the most vulnerable road users because motorcycles offer little physical protection during crashes while exposing riders directly to traffic hazards [2–4]. Across many urban and peri-urban areas of sub-Saharan Africa, commercial motorcycle transport has become an indispensable means of mobility and a major source of employment. However, the rapid expansion of this mode of transport has been accompanied by a substantial increase in motorcycle-related crashes, injuries, and fatalities, posing a growing public health challenge [5]. In Cameroon, motorcycles account for a considerable proportion of road traffic crashes and trauma admissions, particularly among economically productive young adults, underscoring the urgent need for effective and context-specific injury prevention strategies [1, 5].

One of the most important yet frequently overlooked strategies for preventing motorcycle crashes is improving rider conspicuity. Reduced conspicuity is a well-established contributor to motorcycle collisions, particularly during night-time, dawn and dusk, adverse weather conditions, and other situations of poor visibility [6–9]. Compared with larger vehicles, motorcycles are smaller, less visually prominent, and more easily obscured within traffic, increasing the likelihood that other road users fail to detect them in sufficient time to avoid a collision. Visibility-enhancing materials—including reflective jackets or vests, reflective strips mounted on motorcycles, and functional lighting systems such as headlamps, rear lights, brake lights, and indicator lights have consistently been shown to improve motorcycle detection distance and increase the time available for other road users to respond appropriately, thereby reducing crash risk [10, 11]. Consequently, improving rider conspicuity is increasingly recognized as an important component of comprehensive road traffic injury prevention strategies.

Despite these well-documented benefits, the uptake of visibility-enhancing materials among commercial motorcycle riders remains inconsistent across many LMICs because of limited awareness, misconceptions regarding their effectiveness, discomfort, financial constraints, weak enforcement of road safety regulations, and poor access to appropriate safety equipment [12, 13]. Recent evidence from Cameroon similarly demonstrated important deficiencies in riders’ knowledge, attitudes, and visibility-related safety practices. Although approximately half of commercial motorcycle riders reported using reflective jackets, only a small proportion had reflective strips installed on their motorcycles, and fewer than one in ten demonstrated good visibility-related safety practices, indicating substantial gaps between awareness and the consistent adoption of conspicuity measures [13]. These findings suggest that increasing the availability of visibility materials alone is unlikely to produce meaningful behavioural change without interventions that address the behavioural and contextual determinants influencing their use.

Commercial motorcycle transport—commonly known as *okada* transport—plays an indispensable role in Cameroon by providing affordable and accessible transportation, particularly in urban and peri-urban communities where formal public transport systems remain inadequate [5]. In the South-West Region, including the Limbe and Tiko Health Districts, dependence on commercial motorcycles has increased further because of prolonged socio-political instability and economic disruption. Riders frequently operate under conditions characterized by congested roads, mixed traffic, inadequate infrastructure, and poor street lighting, making visibility particularly important for crash prevention. Nevertheless, interventions specifically designed to improve rider conspicuity remain scarce in Cameroon and across much of sub-Saharan Africa. Existing road safety programs in the region have focused predominantly on helmet use, licensing, traffic law enforcement, and general road safety education, with comparatively little emphasis on promoting the routine use of visibility-enhancing materials despite their demonstrated safety benefits.

Evidence from other settings suggests that educational and behavioural interventions can improve selected motorcycle safety behaviours, particularly helmet use and compliance with traffic regulations. However, relatively few interventions have specifically targeted rider conspicuity or systematically evaluated strategies to increase the adoption of visibility-enhancing materials among commercial motorcycle riders, especially in LMICs. Furthermore, most published interventions have not been explicitly guided by behavioural theory or developed using systematic intervention planning frameworks, limiting their reproducibility and potential for adaptation to similar settings.

To address these behavioural determinants, the present intervention was informed by the Health Belief Model (HBM), which proposes that individuals are more likely to adopt protective behaviours when they perceive themselves to be susceptible to injury, recognize the severity of potential consequences, believe that preventive actions are beneficial, perceive barriers to be manageable, receive cues that prompt action, and possess sufficient confidence to perform the behaviour [14]. To translate these theoretical constructs into a practical, contextually relevant intervention, we employed the Intervention Mapping framework, a systematic approach that integrates behavioural theory, empirical evidence, stakeholder engagement, and program planning to develop, implement, and evaluate health promotion interventions [15]. The intervention was further informed by a formative qualitative needs assessment conducted among commercial motorcycle riders and key stakeholders to ensure that intervention components addressed locally identified barriers and facilitators to the uptake of visibility materials.

Although behavioural interventions have shown promise in improving several road safety practices, robust evidence regarding their effectiveness in promoting the sustained use of visibility materials among commercial motorcycle riders in sub-Saharan Africa remains limited. In particular, there is little evidence from Cameroon evaluating theory-informed interventions that combine behavioural science with systematic intervention planning to improve riders’ knowledge, attitudes, and conspicuity-related practices.

Therefore, this study evaluated the effectiveness of a theory-driven health education intervention, developed using the Health Belief Model and the Intervention Mapping framework, in improving knowledge, attitudes, practices, and the use of visibility-enhancing materials among commercial motorcycle riders in the Limbe and Tiko Health Districts of Cameroon. By generating context-specific evidence on behavioural approaches to improving rider conspicuity, this study seeks to inform future road safety programs and contribute to broader injury prevention efforts aimed at reducing motorcycle-related injuries and deaths in Cameroon and other LMICs.

## Materials and methods

### Study design

This study employed a quantitative quasi-experimental, non-randomized controlled before-and-after design to evaluate the effectiveness of a theory-informed health education intervention aimed at improving knowledge, attitudes, and the uptake of visibility materials among commercial motorcycle riders. Limbe Health District was assigned as the intervention district, while Tiko Health District served as the comparison district. Allocation of districts to study arms was based on administrative feasibility and operational considerations rather than randomization. The same cohort of commercial motorcycle riders was followed prospectively from baseline to endline in both districts, allowing assessment of within-group changes over time and estimation of the intervention effect using a difference-in-differences analytical approach. Although formative qualitative methods were employed during the intervention development phase to identify context-specific behavioural determinants and guide intervention design, the present study was designed solely as a quantitative evaluation of the intervention. The study protocol was registered retrospectively with ClinicalTrials.gov (Identifier: NCT07087444; registered July 28, 2025).

### Study setting

The study was conducted in the Limbe and Tiko Health Districts of Fako Division in the South-West Region of Cameroon. Both districts are major urban and peri-urban transport hubs with extensive commercial motorcycle transport activities. The South-West Region has experienced prolonged socio-political instability, resulting in disruptions to public transportation, economic hardship, and increased dependence on commercial motorcycle transport as an important source of livelihood and mobility. Consequently, commercial motorcycle riders in these districts experience frequent exposure to heavy traffic, mixed road use, poor road infrastructure, and night-time riding conditions, placing them at increased risk of road traffic injuries and making them an appropriate population for evaluating interventions aimed at improving rider conspicuity and injury prevention.

### Study population

The study population comprised commercial motorcycle riders operating within the Limbe and Tiko Health Districts. Riders were eligible to participate if they were aged 18 years or older, had operated as commercial motorcycle riders within the study area for at least three months, owned or regularly operated a functional motorcycle, possessed a mobile phone, were able to communicate in either English or Pidgin English, and provided written informed consent. Riders who declined participation or were unable to complete baseline data collection were excluded.

### Sampling and recruitment

Participants for the intervention study were recruited from a previously conducted community-based cross-sectional survey that assessed knowledge, attitudes, and use of visibility materials among commercial motorcycle riders in the Limbe and Tiko Health Districts. The baseline survey was conducted between May 17, 2024, and August 17 2024, during which 499 riders were enrolled, including 300 from Limbe Health District and 199 from Tiko Health District.

At the completion of the baseline survey, participants were informed about a planned follow-up health education intervention designed to improve the use of visibility materials among commercial motorcycle riders. Those willing to participate in the intervention provided their contact information, including mobile telephone numbers, and consented to future follow-up. These riders constituted the sampling frame for the quasi-experimental study.

Eligible participants were subsequently contacted using the telephone numbers provided during the baseline survey and, where necessary, through their respective motorcycle park associations. In the intervention district, 183 riders from the baseline survey were successfully recruited and enrolled, while 130 riders were recruited from the comparison district. Only participants who had completed the baseline survey and consented to follow-up were eligible for inclusion in the intervention study. At endline, 149 participants in the intervention district and 100 participants in the comparison district were successfully followed up and completed the final assessment.

### Sample size considerations

The minimum required sample size was estimated to detect a meaningful improvement in knowledge of visibility materials following the health education intervention. The calculation assumed a baseline knowledge proportion of 32.6%, derived from findings of the baseline cross-sectional survey conducted among commercial motorcycle riders in the study area, an anticipated absolute improvement of 16%, a two-sided confidence level of 95%, and a statistical power of 90%. To account for potential loss to follow-up, a minimum sample of 100 participants per study arm was targeted. Ultimately, 183 riders were enrolled in the intervention district and 130 in the comparison district at baseline, with 149 and 100 participants, respectively, successfully completing the endline assessment.

### Development of the health education intervention

The health education intervention was developed using a systematic, theory-informed, and participatory approach guided by the Health Belief Model (HBM) and the Intervention Mapping framework. The intervention development process was informed by findings from a formative qualitative needs assessment conducted before implementation to identify behavioural, contextual, and environmental factors influencing the uptake of visibility materials among commercial motorcycle riders. Information generated during this formative phase informed the selection of behavioural objectives, educational messages, delivery methods, and implementation strategies to ensure that the intervention addressed barriers and facilitators identified within the local context.

### Formative qualitative needs assessment

Prior to intervention development, a formative qualitative needs assessment was undertaken to identify context-specific behavioural determinants influencing the use of visibility materials among commercial motorcycle riders. Formative qualitative research is conducted before intervention development to improve understanding of the target population, identify locally relevant barriers and facilitators to behaviour change, and ensure that intervention components are contextually appropriate and responsive to participants’ needs.

Four focus group discussions, each comprising six to eight commercial motorcycle riders, were conducted in Limbe Health District using semi-structured discussion guides. Following review of the fourth discussion, no new concepts or issues emerged; therefore, thematic saturation was considered to have been achieved. In addition, key informant interviews were conducted with stakeholders involved in motorcycle transport and road safety, including two experienced commercial motorcycle riders, two motorcycle union leaders, a senior transport authority representative, a road safety police officer, and a healthcare worker involved in injury prevention.

Discussions explored participants’ perceptions of road traffic injury risk, beliefs regarding the importance of rider visibility, barriers and facilitators to the use of reflective jackets and functional motorcycle lighting systems, and potential strategies for improving safety practices among commercial motorcycle riders. All discussions and interviews were audio-recorded with participants’ permission, transcribed verbatim, and analyzed using thematic analysis. Two members of the research team independently reviewed the transcripts, developed initial codes, and grouped similar codes into broader themes through an iterative process. Coding discrepancies were resolved through discussion until consensus was reached. The resulting themes were not analyzed as study outcomes but were used exclusively to identify behavioural determinants and inform the development of intervention objectives, educational content, and delivery strategies within the Intervention Mapping framework.

### Theoretical framework for intervention development

The intervention was grounded in the Health Belief Model, which proposes that individuals are more likely to adopt preventive health behaviours when they perceive themselves to be susceptible to adverse outcomes, recognize the seriousness of those outcomes, believe that the benefits of taking preventive action outweigh potential barriers, receive appropriate cues to action, and possess sufficient confidence to perform the desired behaviour. Accordingly, intervention messages were designed to strengthen riders’ perceptions of susceptibility to road traffic injuries, emphasize the potential severity of motorcycle crashes, highlight the benefits of using visibility materials, address commonly reported barriers to their use, provide regular cues to action, and enhance participants’ confidence in consistently adopting recommended safety practices.

The Intervention Mapping framework was subsequently used to translate findings from the formative needs assessment and behavioural theory into practical intervention strategies. This process involved identifying priority behavioural outcomes, specifying performance objectives, linking behavioural determinants to appropriate theory-based methods, and developing culturally appropriate educational materials tailored to the needs and experiences of commercial motorcycle riders in the study setting.

### Structure and delivery of the intervention

The intervention combined face-to-face health education with mobile phone–based educational reinforcement over an eight-month period. Three interactive group sensitization sessions were conducted in the intervention district: one at the beginning of the intervention, a second session two months later, and a final session two months thereafter. Each session incorporated interactive discussions, demonstrations of appropriate use of reflective jackets and motorcycle lighting systems, clarification of common misconceptions regarding rider visibility, practical problem-solving exercises, and opportunities for participants to share experiences related to road safety and injury prevention.

To reinforce key educational messages between face-to-face sessions, participants received weekly mobile phone–based educational messages throughout the intervention period. Message delivery was tailored to participants’ literacy levels, preferred communication methods, and available technology. Participants with smartphones received weekly WhatsApp text messages accompanied by voice recordings conveying the same content. Participants without smartphones but who were able to read received short message service (SMS) messages, whereas those who preferred verbal communication received scheduled telephone calls during which the educational messages were delivered orally. This multimodal communication strategy ensured equitable access to intervention content regardless of literacy level or mobile phone type.

Attendance registers were maintained during all face-to-face sensitization sessions to monitor participants’ exposure to the intervention.

### Duration of the intervention

The intervention was implemented over an eight-month period, from September 2024 to April 2025.

### Control group

Commercial motorcycle riders in Tiko Health District served as the comparison group. Participants in the comparison district continued to receive the routine road safety information and services ordinarily available within the district but did not receive any study-related education on visibility materials during the intervention period.

### Data collection procedures

Baseline data were collected before implementation of the health education intervention using a structured interviewer-administered questionnaire and a standardized observational checklist. The same data collection procedures were repeated at the end of the eight-month intervention period to evaluate changes in study outcomes in both the intervention and comparison districts.

The questionnaire was developed from an extensive review of the literature and findings from the formative qualitative needs assessment. It comprised four sections assessing participants’ socio-demographic characteristics, knowledge of visibility materials, attitudes towards their use, and self-reported visibility-related practices. Knowledge was assessed using multiple-choice and dichotomous questions covering the purpose, types, and appropriate use of visibility materials. Attitudes were assessed using statements based on the constructs of the Health Belief Model, while practices assessed the routine use and maintenance of visibility-enhancing materials during commercial motorcycle operations.

In addition to the questionnaire, trained research assistants conducted direct observations to objectively verify the presence and functionality of visibility materials on participants’ motorcycles and the use of reflective jackets. Immediately after completion of each interview, participants were accompanied to their motorcycles, where research assistants completed a standardized observational checklist documenting the presence and condition of reflective jackets, reflective strips, headlights, brake lights, rear lights, and indicator lights. The same observation procedures, instruments, and assessment criteria were used at baseline and endline in both the intervention and comparison districts to ensure consistency of outcome measurement.

The questionnaire and observational checklist were pretested among commercial motorcycle riders in a neighbouring health district that was not included in the study. Feedback obtained during the pretest was used to improve the clarity, sequencing, and comprehensibility of questionnaire items before commencement of data collection. Data collectors received standardized training on study procedures, interview techniques, observational assessments, ethical conduct, and quality assurance to ensure uniform implementation of the study protocol across both districts.

Completed questionnaires were reviewed daily by field supervisors for completeness, internal consistency, and accuracy. Where necessary, incomplete questionnaires were corrected immediately while participants were still available. Data were subsequently entered into a secure electronic database using double-entry procedures to minimize transcription errors, and regular data quality checks were performed throughout the study period.

### Study outcomes

The primary outcome was the uptake of visibility materials among commercial motorcycle riders following implementation of the health education intervention. Uptake was assessed through both self-reported use and direct observation of reflective jackets, reflective strips, and functional motorcycle lighting systems.

Secondary outcomes included changes in participants’ knowledge, attitudes, and visibility-related safety practices. Knowledge scores were calculated by assigning one point to each correct response and zero points to incorrect or “don’t know” responses. Attitude scores were derived from participants’ responses to Likert-scale statements measuring perceptions related to susceptibility, severity, benefits, barriers, cues to action, and self-efficacy regarding the use of visibility materials. Practice scores reflected participants’ reported routine use and maintenance of visibility-enhancing materials during commercial motorcycle operations.

Composite scores for knowledge, attitudes, and practices were generated using predefined scoring criteria consistent with the baseline survey. Participants were subsequently categorized into appropriate outcome groups for analysis based on these established cut-off values.

### Strategies to minimize bias

Several measures were implemented to enhance the internal validity of the study and reduce potential sources of bias. First, identical eligibility criteria, recruitment procedures, data collection instruments, and assessment methods were used in both the intervention and comparison districts. Second, the same standardized training was provided to all research assistants before baseline and endline data collection to ensure consistency in interviewing and observational procedures. Third, objective direct observations were conducted alongside self-reported questionnaire data to reduce the likelihood of information bias associated with self-reporting. Fourth, data quality was maintained through daily supervision, routine review of completed questionnaires, double data entry, and periodic verification of electronic data. Finally, advanced analytical approaches, including generalized estimating equations and difference-in-differences analysis, were used to account for repeated measurements and estimate the intervention effect while minimizing the influence of measured baseline differences between study groups.

### Statistical analysis

All analyses were conducted using SPSS version 26. Descriptive statistics were used to summarize participant characteristics and outcome measures at baseline and endline. Attrition analyses were conducted separately for the intervention and control groups to compare baseline socio-demographic characteristics between participants retained at endline and those lost to follow-up using chi-square tests.

Within-group changes between baseline and endline were examined separately for the intervention and control groups. Paired samples t-tests were used to evaluate changes in continuous outcomes such as knowledge and attitude scores, while McNemar’s tests were used for binary outcomes related to the use of reflective jackets and functional lighting systems.

The primary evaluation of the intervention effect was conducted using a difference-in-differences approach implemented through generalized estimating equation models to account for repeated measurements over time. Logistic GEE models with a logit link were fitted for binary outcomes, while linear GEE models were used for continuous outcomes. Each model included study group, time, and a group-by-time interaction term representing the intervention effect. Both unadjusted and adjusted models were fitted. Adjusted models controlled for age group, marital status, education level, and years of riding experience. Adjusted odds ratios or regression coefficients with 95% confidence intervals were reported, and statistical significance was assessed at *p* < 0.05. Knowledge, attitude and practice scores were treated as continuous variables in all analyses and were not categorized, in order to preserve statistical power and avoid information loss.

### Post-intervention access for the control group

After completion of endline data collection, riders in the control district were offered access to the health education messages using the same mobile phone–based delivery approaches for a period of two months to ensure fairness and ethical balance.

## Results

### Baseline socio-demographic characteristics of study participants by study group

A total of 183 riders were enrolled in the intervention Health District (Limbe) at baseline and 149 were retained at endline. A total of 130 riders were enrolled at baseline in the controlled Health District (Tiko) and 100 were retained at the end of the intervention. A total of 249 commercial motorcycle riders were therefore retained at the end of the health education intervention study comprising 149 riders from Limbe Health District and 100 riders from Tiko Health District. The mean age of riders in the Limbe and Tiko Health Districts were 33.4 ± 7.3 years and 32.0 ± 7.8 years respectively. All riders in this study were males and most riders in both health districts were aged 31–40 years, accounting for 43.6% in Limbe and 39.0% in Tiko. Overall, the age distribution of riders was broadly similar in both health districts, with the majority being younger adults (Table 1).

**Table 1:** Socio-demographic characteristics of retained riders in the control and intervention groups (*n* = 249)

		Health District		
Variable	Category	Limbe	Tiko	Total
Age group (years)	41–50	26(16.8)	11(8)	33(13.3)
	31–40	65(43.6)	39(39)	104(41.8)
	21–30	58(38.9)	50(50)	108(43.4)
Marital status	Married	80(53.7)	38(38)	118(47.4)
	Single	69(45.3)	62(62)	126(50.6)
Highest education	Tertiary	14(9.4)	5(5)	19(7.6)
	Secondary	81(54.4)	61(61)	142(57)
	Primary	43(28.9)	30(30)	73(29.3)
	No formal	11(7.4)	4(4)	15(6)
Average monthly income (XAF1000)	> 150	11(7.4)	8(8)	19(7.6)
	101–150	47(31.5)	27(27)	74(29.7)
	50–100	85(57)	61(61)	146(58.6)
	< 50	6(4)	4(4)	10(4)
Riding duration (years)	> 10	26(17.4)	17(17)	43(17.3)
	5–10	48(32.2)	29(29)	77(30.9)
	< 5	75(50.3)	54(54)	129(51.8)

### Assessment of loss to follow-up (attrition analysis) in the control and intervention groups

An attrition analysis was conducted to assess whether riders lost to follow-up differed systematically from those retained at endline in the intervention group (Limbe Health District) and control group (Tiko Health District). Baseline characteristics of riders who were retained and those lost to follow-up were compared using chi-square tests.

There was a statistically significant difference in attrition by age group (*p* = 0.029), indicating that loss to follow-up varied across age categories. Specifically, participants within the age group > 50 were all lost to follow up. However, no statistically significant differences were observed between retained and lost participants with respect to marital status (*p* = 0.989), level of education (*p* = 0.392) or years of riding (*p* = 0.663) (Table 2).

Overall, apart from age group, baseline socio-demographic characteristics were comparable between riders retained at endline and those lost to follow-up in Limbe.

**Table 2:** Baseline characteristics of riders retained and lost to follow-up in Limbe Health District

Variable	Category	Lost to follow-up *n* (%)	Retained at endline *n* (%)	*p*-value
Age group (years)	>50	3(100.0)	0(0.0)	0.029
	41–50	0 (0.0)	25 (100.0)	
	31–40	16 (19.8)	65 (80.2)	
	21–30	18(23.7)	58 (76.3)	
Marital status	Married	18 (18.4)	80 (81.6)	0.989
	Single	16 (19.8)	69 (80.2)	
Education level	Tertiary	4 (22.2)	14 (77.8)	0.392
	Secondary	21 (20.6)	81 (79.4)	
	Primary	9 (17.3)	43 (82.7)	
	No formal	0 (0.0)	11 (100.0)	
Years of riding	> 10	4 (13.3)	26 (86.7)	0.663
	5–10	12 (20.0)	48 (80.0)	
	< 5	18 (19.4)	75 (80.6)	

Percentages are calculated within each category

In the Tiko health district, there was a statistically significant association between age group and attrition (*p* < 0.001), indicating that loss to follow-up differed across age categories. However, no statistically significant differences were observed between retained and lost participants with respect to marital status (*p* = 0.225), level of education (*p* = 0.980), average monthly income (*p* = 0.720), or years of riding (*p* = 0.148). Apart from age group, baseline characteristics were comparable between riders retained at endline and those lost to follow-up in Tiko (Table 3).

**Table 3:** Baseline characteristics of riders retained and lost to follow-up in Tiko Health District

Variable	Category	Lost to follow-up *n* (%)	Retained at endline *n* (%)	*p*-value
Age group (years)	> 50	5 (100.0)	0(0.0)	<0.001
	41–50	10 (55.6)	8 (44.4)	
	31–40	14 (25.9)	40 (74.1)	
	21–30	4 (7.5)	49 (92.5)	
Marital status	Married	14 (26.4)	39 (73.6)	
	Single	16 (20.0)	61 (80.0)	
Highest education level	Tertiary	2 (28.6)	5 (71.4)	0.98
	Secondary	16 (21.1)	60 (78.9)	
	Primary	11 (26.2)	31 (73.8)	
	No formal	1 (20.0)	4 (80.0)	
Average monthly income (in 1000XAF)	> 150	3 (27.3)	8 (72.7)	0.72
	101–150	11 (28.9)	27 (71.1)	
	50–100	15 (19.7)	61 (80.3)	
	< 50	1 (20.0)	4 (80.0)	
Years of riding (years)	> 10	9 (36.0)	16 (64.0)	0.148
	5–10	10 (25.0)	30 (75.0)	
	< 5	11 (16.9)	54 (83.1)	

Percentages are calculated within each category / row

### Within-group comparisons between baseline and endline

#### Within-group comparison of KAP scores in the control group (Tiko Health District)

Paired samples t-tests were conducted to compare baseline and endline scores for knowledge, attitudes and practices related to visibility materials among riders in the control group-Tiko Health District (Table 4). There were no statistically significant differences between baseline and endline scores for knowledge (mean difference = 0.07, t = 0.385, *p* = 0.701), attitudes (mean difference = 0.17, t = 0.430, *p* = 0.668), or practices (mean difference = − 0.13, t = 0.647, *p* = 0.519).

Overall, these findings indicate that knowledge, attitudes and practices related to visibility materials, did not change significantly.

**Table 4:** Within-group comparison of KAP scores in the control group (Tiko Health District)

Outcome	Mean difference (Endline–Baseline)	t	*p*-value
Visibility material knowledge	0.07	0.385	0.701
Visibility material attitude	0.17	0.43	0.668
Visibility material practice	0.13	0.647	0.519

*Paired t-test* mean difference represents endline minus baseline scores

#### Within-group comparison of visibility materials use at baseline and endline among riders in Tiko Health District

McNemar’s test was used to assess changes in the use of visibility materials between baseline and endline among commercial motorcycle riders in the control group-Tiko Health District (Table 5). There was no statistically significant change in the use of any of the visibility materials assessed between baseline and endline. Overall, these findings indicate that the use of visibility materials among riders in the control group did not change significantly over time.

**Table 5:** Within-group comparison of visibility materials use at baseline and endline among riders in Tiko Health District (*n* = 100)

Visibility materials	Baseline Use (%)	Endline Use (%)	% Change (Endline-Baseline)	*p*-value
Backlight	72.0	79.6	7.6	0.883
Jacket	11.0	15.5	4.5	0.324
Strips	84.0	88.8	4.8	0.424
Lamp	47.0	49.5	2.5	0.523
Brake light	55.0	53.5	-1.5	0.883
Trafficator	35.0	32.3	-2.7	0.775

Percentages (%) are based on *n* = 100 riders

*p*-values were obtained using McNemar’s test

#### Within-group comparison of KAP scores in the intervention group (Limbe Health District)

There were statistically significant increases in knowledge scores (mean difference = 1.75, t = 8.93, *p* < 0.001), attitude scores (mean difference = 5.60, t = 10.67, *p* < 0.001), and practice scores (mean difference = 0.36, t = 2.55, *p* = 0.012). Overall, these findings indicate that knowledge, attitudes and practices related to both visibility materials improved significantly over time among riders in the intervention group (Table 6).

**Table 6:** Within-group comparison of KAP scores in the intervention group (Limbe Health District)

Outcome	Mean difference (Baseline – Endline)	t	*p*-value
Visibility material knowledge	1.75	8.93	<0.001
Visibility material attitude	5.6	10.67	<0.001
Visibility material practice	0.36	2.55	0.012

*Paired t-test* mean difference represents endline minus baseline scores

#### Within-group comparison of visibility materials use in the intervention group (Limbe Health District)

McNemar’s test was used to assess changes in the use of visibility materials between baseline and endline among commercial motorcycle riders in the intervention group-Limbe Health District (Table 7). There were statistically significant increases in the use of reflective jackets (*p* < 0.001), backlights (*p* = 0.004), brake lights (*p* = 0.023), and trafficators/indicator lights (*p* = 0.004) between baseline and endline. A borderline statistically significant increase in lamp use was also observed (*p* = 0.050). In contrast, there was no statistically significant change in the use of reflective strips between baseline and endline (*p* = 1.000). Overall, these findings indicate that the health education intervention was associated with significant improvements in the use of most visibility materials among riders in the intervention group, with the exception of reflective strips, for which no significant change was observed.

**Table 7:** Within-group comparison of visibility materials use at baseline and endline among riders in Limbe Health District (*n* = 149)

Visibility material	Baseline use (%)	Endline Use (%)	(%) Change (Endline-Baseline)	*p*-value
Reflective jacket	72.0	79.6	7.6	<0.001
Reflective strips	11.0	15.5	0.7	1.000
Lamp	84.0	88.8	8.1	0.050
Backlight	47.0	49.5	6.0	0.004
Brake light	47.0	53.5	6.5	0.023
Trafficator (indicator)	35.0	32.3	6.0	0.004

### Difference-in-differences analysis of the impact of the health education intervention

To estimate the causal effect of the health education intervention on the use of visibility materials, a difference-in-differences (DiD) analytical approach was employed. The DiD method compares changes in outcomes over time in the intervention group (Limbe Health District) with changes over the same period in a comparable control group (Tiko Health District). This approach allows the effect of the intervention to be isolated from background temporal changes and other external influences that may affect both groups simultaneously, such as seasonal variations, enforcement activities or general public awareness activities.

#### Unadjusted difference-in-differences analysis of visibility materials use

Unadjusted difference-in-differences analyses using generalized estimating equations were conducted to assess the effect of the health education intervention on the use of visibility materials (Table 8). The intervention was associated with a statistically significant increase in reflective jacket use (COR = 4.21, 95% CI: 1.84–9.64, *p* = 0.001). No statistically significant intervention effects were observed for reflective strips use, headlamp use, backlight use, brake light use, or trafficator (indicator) light use (all *p* > 0.05).

The unadjusted models suggest that the health education intervention significantly improved reflective jacket use among commercial motorcycle riders, while no significant effects were observed for the other PPE and visibility materials.

**Table 8:** Unadjusted difference-in-differences (GEE) estimates of the effect of the intervention on PPE and visibility materials use

Outcome		95% CI for COR		
	COR (Group x Time)	Lower	Upper	*p* value
Jacket use	4.210	1.838	9.641	0.001
Strips use	0.756	0.240	2.378	0.632
Headlamp use	1.408	0.505	3.930	0.513
Backlight use	1.146	0.653	2.010	0.635
Brake light use	0.620	0.312	1.231	0.172
Four trafficator (indicator) lights use	1.415	0.748	2.676	0.286

*COR  *crude odds ratio from GEE logistic difference-in-differences models. The intervention effect is represented by the Group × Time interaction term

#### Adjusted difference-in-differences analysis of PPE and visibility materials use

After adjusting for age group, level of education, average monthly income and riding experience, the health education intervention was associated with a statistically significant increase reflective jacket use (AOR = 4.34, 95% CI: 1.88–10.02, *p* = 0.001). No statistically significant intervention effects were observed for reflective strips use, headlamp use, backlight use, brake light use or trafficator (indicator) light use (all *p* > 0.05).

Overall, the adjusted models indicate that the health education intervention significantly improved reflective jacket use among commercial motorcycle riders, while no significant effects were observed for the other visibility materials assessed (Table 9).

**Table 9:** Adjusted difference-in-differences analysis of PPE and visibility materials use

Outcome	AOR (Group × Time)	95% CI	*p*-value
Reflective jacket use	4.34	1.88–10.02	0.001
Reflective strips use	0.75	0.23–2.44	0.634
Headlamp use	1.35	0.56–3.24	0.501
Backlight use	1.15	0.65–2.06	0.632
Brake light use	0.61	0.30–1.24	0.173
Trafficator (indicator) light use	1.45	0.74–2.83	0.282

*AOR  *adjusted odds ratio from GEE logistic difference-in-differences models. Models were adjusted for age group, level of education, average monthly income and riding experience. The intervention effect is represented by the Group × Time interaction term

### Difference-in-differences analysis for continuous outcomes

#### Unadjusted difference-in-differences analysis of KAP scores

Unadjusted difference-in-differences analyses using generalized estimating equations were conducted to assess the effect of the health education intervention on knowledge, attitudes and practices related to personal protective equipment and visibility materials. The intervention was associated with statistically significant increases in PPE knowledge scores (β = 2.91, 95% CI: 2.14–3.68, *p* < 0.001) and PPE attitude scores (β = 5.76, 95% CI: 4.32–7.21, *p* < 0.001). No statistically significant intervention effect was observed for PPE practice scores (β = 0.21, 95% CI: −0.09–0.52, *p* = 0.171). Similarly, statistically significant increases were observed in visibility materials knowledge scores (β = 1.68, 95% CI: 1.16–2.20, *p* < 0.001) and visibility materials attitude scores (β = 5.51, 95% CI: 4.22–6.80, *p* < 0.001). However, no statistically significant intervention effect was found for visibility materials practice scores (β = 0.34, 95% CI: −0.11–0.79, *p* = 0.141). Overall, the unadjusted models indicate that the health education intervention significantly improved knowledge and attitudes related to both PPE and visibility materials, whereas improvements in practices were not statistically significant.

#### Adjusted difference-in-differences analysis of KAP scores

After adjusting for age group, marital status, level of education and years of riding, the health education intervention was associated with statistically significant improvements in knowledge and attitudes related to both personal protective equipment (PPE) and visibility materials (Table 10).

Specifically, PPE knowledge scores increased significantly in the intervention group compared with the control group (β = 2.91, 95% CI: 2.14–3.68, *p* < 0.001), as did PPE attitude scores (β = 5.76, 95% CI: 4.32–7.21, *p* < 0.001). However, no statistically significant intervention effect was observed for PPE practice scores (β = 0.21, 95% CI: −0.09–0.52, *p* = 0.171). Similarly, visibility materials knowledge (β = 1.68, 95% CI: 1.16–2.20, *p* < 0.001) and visibility materials attitude scores (β = 5.51, 95% CI: 4.22–6.80, *p* < 0.001) improved significantly following the intervention, whereas no statistically significant change was observed in visibility materials practice scores (β = 0.34, 95% CI: −0.11–0.79, *p* = 0.141). These findings indicate that the health education intervention significantly improved riders’ knowledge and attitudes toward PPE and visibility materials but did not produce statistically significant changes in reported practices.

The similarity between the unadjusted and adjusted difference-in-differences estimates indicates that the measured socio-demographic variables did not confound the relationship between the health education intervention and the outcomes. The intervention effect therefore appears to be robust to adjustment for age, marital status, education and years of riding.

**Table 10:** Unadjusted difference-in-differences (GEE) estimates of the effect of the intervention on KAP scores

Outcome	β (Group × Time)	95% CI	*p*-value
Visibility materials knowledge score	1.68	1.16–2.20	<0.001
Visibility materials attitude score	5.51	4.22–6.80	<0.001
Visibility materials practice score	0.34	−0.11–0.79	0.141

All models adjusted for age group, marital status, education and years of riding

β represents the difference-in-differences estimate from linear GEE models.The intervention effect is represented by the Group × Time interaction term

## Discussion

This quasi-experimental study evaluated the effectiveness of a theory-informed health education intervention in improving knowledge, attitudes, and the use of visibility materials among commercial motorcycle riders in Cameroon. Overall, the findings demonstrate that the intervention significantly improved riders’ knowledge and attitudes towards visibility materials and resulted in a meaningful increase in reflective jacket use. In contrast, no significant improvements were observed in the comparison district, suggesting that the observed changes were attributable to the intervention rather than to secular trends or external influences. Although improvements were also observed in the use of backlights, brake lights, and indicator lights within the intervention district, these changes were not statistically significant after adjustment for potential confounders. Collectively, these findings indicate that while educational interventions can effectively influence cognitive determinants of behaviour, translating these gains into sustained adoption of some visibility materials may require complementary structural and policy interventions.

One of the principal findings of this study was the significant improvement in riders’ knowledge and attitudes towards visibility materials following the intervention. These findings are consistent with the Health Belief Model, which posits that individuals are more likely to adopt preventive behaviours when they perceive themselves to be susceptible to adverse outcomes, recognize the seriousness of those outcomes, appreciate the benefits of preventive actions, and believe they are capable of performing the desired behaviour [14]. The intervention addressed these constructs through interactive health education sessions that highlighted the burden of motorcycle-related injuries, emphasised the protective role of visibility materials, addressed common misconceptions, and provided repeated reinforcement through mobile phone reminders. The observed improvements therefore suggest that the intervention successfully modified riders’ perceptions of risk and strengthened their motivation to adopt safer visibility practices. Similar findings have been reported in previous theory-based educational interventions, which have demonstrated that behavioural models can improve safety-related knowledge, attitudes, and behavioural intentions among vulnerable populations [16–19].

The effectiveness of the intervention may also be attributed to its systematic development using the Intervention Mapping framework. Unlike conventional health education programs that focus primarily on information dissemination, Intervention Mapping provides a structured process for linking behavioural theory with empirical evidence and local context to develop interventions that address identified determinants of behaviour [15]. In the present study, the intervention was informed by a formative qualitative needs assessment that identified poor risk perception, misconceptions regarding reflective materials, and weak social norms supporting conspicuity as important barriers to the use of visibility materials. These findings informed the development of culturally appropriate educational messages, practical demonstrations, and mobile phone–based reinforcement that were responsive to the needs of commercial motorcycle riders. The significant improvements in knowledge and attitudes observed in this study highlight the importance of combining behavioural theory with context-specific intervention planning to enhance intervention relevance, acceptability, and effectiveness.

Although knowledge and attitudes improved substantially, reflective jacket use was the only visibility-related behaviour that remained significantly associated with the intervention after adjustment for baseline differences and secular trends. This finding illustrates an important principle in behaviour change research: improvements in knowledge and attitudes do not necessarily translate into immediate adoption of all recommended behaviours [16]. Reflective jackets may have been more readily adopted because they are relatively inexpensive, portable, and easy to use without requiring modifications to the motorcycle [17]. Furthermore, the repeated demonstrations during group sensitization sessions and weekly mobile phone reminders likely served as effective cues to action that reinforced daily use. From an injury prevention perspective, this finding is particularly important. Within the Haddon Matrix, reflective clothing is considered a pre-event intervention that improves rider conspicuity and reduces the likelihood of collisions by increasing motorcycle visibility before a crash occurs. Likewise, the Safe System Approach recognizes conspicuity-enhancing measures as important components of comprehensive road safety strategies that acknowledge human error while seeking to minimize crash occurrence and injury severity. The observed increase in reflective jacket use therefore extends beyond behavioural change and demonstrates the potential contribution of visibility-enhancing interventions to broader motorcycle injury prevention efforts.

In contrast, the absence of statistically significant intervention effects for reflective strips and motorcycle lighting systems suggests that educational interventions alone may be insufficient to overcome barriers associated with these behaviours. During the formative needs assessment, participants identified financial constraints, poor access to quality replacement parts, inadequate enforcement of road safety regulations, and challenges related to motorcycle maintenance as important barriers to maintaining functional lighting systems and installing reflective strips [20]. These barriers are largely structural and may not be addressed through education alone. Consequently, while behavioural interventions remain essential for improving awareness and motivation, they should be complemented by supportive environmental and policy measures, including improved enforcement of visibility-related regulations, increased availability of affordable visibility equipment, and partnerships with commercial motorcycle unions and repair workshops to facilitate maintenance of safety equipment. Such multilevel approaches are more likely to achieve sustained improvements in visibility-related practices.

An important strength of this study is the consistency of findings across multiple analytical approaches. The absence of significant changes in the comparison district, together with the significant intervention effects observed using both difference-in-differences and generalized estimating equation analyses, strengthens confidence that the observed improvements resulted from the intervention rather than from external influences or underlying temporal trends. Furthermore, the intervention was developed through a systematic, theory-informed process and evaluated using a quasi-experimental design with repeated measurements, thereby providing stronger evidence than cross-sectional studies that have traditionally dominated research on motorcycle safety behaviours in sub-Saharan Africa. By specifically focusing on visibility materials rather than helmet use alone, this study also contributes to an emerging area of motorcycle injury prevention research that has received comparatively limited attention despite the recognized importance of rider conspicuity in reducing crash risk [21–25].

Several limitations should be considered when interpreting these findings. First, the quasi-experimental design does not provide the same level of causal inference as a randomized controlled trial, although the use of a comparison district and robust analytical methods reduced the potential for bias. Second, although direct observation was used to verify the presence and functionality of several visibility materials, some practice-related measures relied on self-report and may therefore have been influenced by recall or social desirability bias. Third, the study evaluated outcomes over an eight-month period, and longer-term follow-up is needed to determine whether the observed behavioural improvements can be sustained after completion of the intervention. Finally, the study was conducted in two health districts within one region of Cameroon, which may limit the generalisability of the findings to other settings with different road environments or regulatory contexts.

In conclusion, this study demonstrates that a health education intervention systematically developed through the Intervention Mapping framework and guided by the Health Belief Model can significantly improve visibility-related knowledge, attitudes, and reflective jacket use among commercial motorcycle riders. However, achieving broader improvements in the use of other visibility materials will likely require educational interventions to be complemented by structural and policy measures that address financial, technical, and environmental barriers. Integrating behavioural education with strengthened road safety enforcement, improved access to affordable visibility equipment, and active engagement of commercial motorcycle unions may provide a practical and sustainable strategy for reducing motorcycle-related injuries in Cameroon and other low- and middle-income countries.

## Conclusion

This study shows that a health education intervention developed through the Intervention Mapping approach and guided by the Health Belief Model significantly improved commercial motorcycle riders’ knowledge and attitudes toward visibility materials in Limbe Health District. The intervention also produced a significant increase in reflective jacket use, indicating improved conspicuity behaviour among riders. However, no significant intervention effects were observed for most motorcycle lighting and reflective components, including reflective strips and functional lighting systems. These findings indicate that theory-based educational interventions can enhance awareness and motivation to use visibility materials but may be insufficient on their own to overcome structural and resource-related constraints affecting the adoption of certain visibility devices. Integrating educational strategies with broader regulatory, enforcement and access-oriented measures is likely necessary to achieve sustained improvements in rider visibility and injury prevention. These findings are relevant to other low- and middle-income settings where commercial motorcycle transport is common and road safety systems are underdeveloped.

## Supplementary Information

Additional file 1: Structured questionnaire used for data collection (English version).

## Data Availability

All data supporting the findings of this study are available within the paper and its Supplementary Information.
